# Novel technology for non-invasive thoracic fluid measurement: an animal model comparative study

**DOI:** 10.1186/cc13327

**Published:** 2014-03-17

**Authors:** G Karp, M Jonas, T Mandelbaum, E Kishinevsky, N Adi

**Affiliations:** 1 Kaplan Medical Center, Rehovot, Israel; 2 Sheba Medical Center, Tel Aviv, Israel

## Introduction

Approximately 15 million people worldwide suffer from congestive heart failure (CHF). The most severe symptom of CHF is pulmonary congestion - an acute increase in extravascular lung fluid due to acute decompensation of heart failure. To date, no direct, reliable, simple and non-invasive method is available for assessment of thoracic fluids. Continuous dependable monitoring of pulmonary edema for hospital patients can improve management and reduce duration of hospitalization. KYMA's solution is based on a matchboxsized monitoring device, which monitors thoracic fluid content and trends. The μCor monitor transmits and receives electromagnetic signals that are propagated through tissue layers. Conduction is highly related to the amount of accumulated fluids in tissues. This trial investigated the correlation between KYMA's lung water index (LWI) and directly assessed extravascular lung water (EVLW) in the scenario of acute pulmonary edema induced in seven sheep.

## Methods

Acute pulmonary edema was induced by intravenous infusion of noradrenaline and dextrane, with stepwise increased dosage. KYMA measurements of LWI were compared with PiCCO extravascular lung volume as the reference gold standard. The experiment continued until maximum increase of EVLW and complete heart failure were reached.

## Results

All seven sheep developed pulmonary edema, which was validated by increased LVEDP and EVLW. A linear correlation was found between invasive measurements of EVLW (PiCCO) and non-invasive KLWI.

## Conclusion

**Figure 1:**
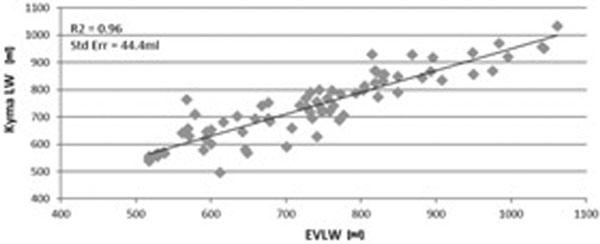
**Pooled correlation between EVLW and KYMA LWI**.

KYMA's fluid index demonstrated excellent correlation with invasive lung water measurement (*R*^2 ^= 0.96; *P *< 0.0001) and was able to detect dynamic accumulation of EVLW in a range of 40 to 50 cm^3 ^increments (Figure 1). Since the change in fluid content between a normal and congested lung ranges between 250 and 500 cm^3^, the demonstrated sensitivity as reflected in this study, supports its use for high-resolution and accurate thoracic fluid monitoring.

